# Seroprevalence and risk factors for hepatitis C virus (HCV) infection in pregnant women attending public sector tertiary care hospital in Hyderabad Sindh

**DOI:** 10.12669/pjms.292.3211

**Published:** 2013-04

**Authors:** Seema Bibi, Saira Dars, Sanober Ashfaq, Roshan Ara Qazi, Sadaf Akhund

**Affiliations:** 1 Dr. Seema Bibi, FCPS(Obs/Gyn), Associate Professor, Department of Obstetrics/Gynaecology, Liaquat University of Medical & Health Sciences Jamshoro, Sindh, Pakistan.; 2 Dr. Saira Dars, MS(Obs/Gyn), Consultant Gynaecologist, Department of Obstetrics/Gynaecology, Liaquat University Hospital Hyderabad/Jamshoro, Sindh, Pakistan.; 3 Dr. Sanober Ashfaq, MS(Obs/Gyn), Registrar, Department of Obstetrics/Gynaecology, Liaquat University Hospital Hyderabad/Jamshoro, Sindh, Pakistan.; 4 Roshan Ara Qazi, FCPS(Obs/Gyn), Chairperson/Professor, Department of Obstetrics/Gynaecology, Liaquat University of Medical & Health Sciences Jamshoro, Sindh, Pakistan.; 5 Dr. Sadaf Akhund, DGO, Trainee Registrar, Department of Obstetrics/Gynaecology, Liaquat University of Medical & Health Sciences Jamshoro, Sindh, Pakistan.

**Keywords:** Hepatitis C infection, Pregnancy, Risk factors, Seroprevalence

## Abstract

***Background and Objectives:*** Pakistan is among the countries having high prevalence of HCV infection in the population but there is dearth of proper epidemiological data regarding acquisition of HCV infection in the pregnant population. Our objective was to determine the seroprevalence of HCV antibodies in healthy pregnant women and to assess the potential risk factors for HCV infection in HCV positive subjects and in the control group.

***Methodology: ***This cross sectional and comparative study was conducted from 1^st^ January to 31^st^ December 2010 in the Department of Obstetrics/Gynaecology Unit–I, Liaquat University Hospital Hyderabad. Sera were collected from all admitted pregnant women and tested for HCV anti bodies using Elisa kits (Abbott, USA). Data were analyzed using SPSS version 16.0 statistical package.

***Results:*** The seroprevalence of HCV among pregnant population was found to be 4.7%. HCV positive women were more likely to have a history of blood transfusion (OR 1.99, 95% CI 1.26- 3.12), History of therapeutic injection use (OR 2.46, 95% CI 1.43-4.26), history of surgery (OR 1.72, 95% CI 1.12-2.66) and history of sharing household products (OR 1.81, 95% CI 1.14-2.87).

***Conclusion:*** HCV seropositive pregnant women were more likely to have a history of blood transfusion, therapeutic injection use, surgery and sharing household items.

## INTRODUCTION

Hepatitis C virus is one of the common blood borne infections worldwide. According to the World Health Organization (WHO) about 3% of the global population i.e. 170 million people are infected by Hepatitis C virus^1^ and is responsible for deadly consequences like cirrhosis and liver cancer. The prevalence of HCV varies epidemiologically with reported prevalence of less than 1% in western countries and greater than 2% in African & Asian countries.^2^ A recent review of available data from Pakistan revealed HCV prevalence as 3% in the general population. A wide frequency of HCV seroprevalence was reported in the pregnant population, ranging from 3.3% to 29.1% with overall frequency of 7.3%.^3^ Overall, HCV infection is not associated with significant harmful effects on mother and fetus, although it is found to be associated with cholestasis of pregnancy, preterm delivery, intrauterine growth restriction and fetal infection.

Worldwide, the well established risk factors for acquisition of HCV infection among pregnant population include intravenous (I/V) drug abuse, history of blood transfusion, hepatitis B virus (HBV) and human immunodeficiency virus (HIV) infection, history of multiple sexually transmitted infections (STIs), multiple sexual partners and sexual contacts with I/V drug users.^4^ In Pakistan, there is a paucity of data on this important public health problem particularly in pregnant women. We routinely screen all pregnant women for HCV infection. We therefore carried out this study to determine the seroprevalence of HCV infection as well as to analyze the risk factors for acquisition of this infection among the obstetric population. The data will help policy makers to establish the burden of disease in the pregnant population and will assist in the planning and prioritizing of preventive strategies particularly in rural areas.

## METHODOLOGY

This study was conducted in the Department of Obstetrics and Gynaecology Unit-I, Liaquat University Hospital (LUH), Hyderabad between 1^st^ January 2010 to 31^st^ December 2010. This hospital is one of the oldest and busiest hospitals of Sindh Province catering for a large number of both urban and rural populations. This cross sectional comparative survey utilized simple, convenient sampling technique for recruitment. The calculated sample size was 343, by taking the frequency of HCV seropositivity in pregnant women as 29.1%.^5^ Study participants included pregnant women who were admitted in the Department for various obstetrical reasons including delivery. After informed consent, about 5mls of blood sample was collected from each participant and tested for HCV antibodies on Elisa (AXSYM-ABBOTT, USA) in the Research & Diagnostic Laboratory of Liaquat University of Medical & Health Sciences, Jamshoro. Both cases and controls were evaluated by detailed history including demographics, obstetrical record and potential risk factors for acquisition of HCV infection. The data was noted down on predesigned performa by the principal author and her team.

The risk factors which were assessed among cases & controls included history of blood transfusion, therapeutic injections, surgery including dilatation and curettage, Caesarean delivery and laparotomy, dental consultations, extramarital sexual relations in women and their husbands, intravenous drug abuse, ear and nose piercing and tattooing, HCV infection in family members and sharing of house hold items.

Results were analyzed using SPSS version 16 statistical package. Odds ratio (OR) and 95% confidence interval were calculated to measure the association between cases and controls with regard to risk factors chosen for study.

Multivariate logistic regression analyses was done to calculate adjusted odds ratio and 95% confidence interval to see the direct effect of single risk factor on HCV positive status. P-value of less than 0.05 was considered significant.

**Table-I:** Demographic details of the women (n =343

	*Positive* *n = 146*	*Negative* *n = 197*	*Total*	*P - Value*
Age	28.8+4.21	28.6+5.02	28.7 + 4.9	0.65
Gravidity	4.23+2.78	4.19+2.65	4.2 + 2.7	0.90
Para	3.00+2.93	2.95+2.84	2.9 + 2.8	0.85
Gestational age in weeks	34.62+4.55	33.76+4.81	34.12 + 4.7	0.09
Weight of baby	2.76+0.44	2.78+0.41	2.7 + 0.4	0.71
Apgar score in 5 minute	7.47+1.73	7.58+1.57	7.5 + 0.42	0.56
*Booked/Unbooked*				
Booked	54(37.0%)	60(30.5%)	114(33.2%)	0.24
Unbooked	92(63.0%)	137(69.5%)	229(66.8%)	
*Occupation*				
Housewife	127(87.0%)	178(90.4%)	305(88.9%)	0.38
Working	19(13.0%)	19(9.6%)	38(11.1%)	
*Monthly income *				
< 6000	79(54.1%)	83(42.1%)	162(47.2%)	0.02*
> 6000	67(45.9%)	114(57.9%)	181(52.8%)

*P value is statistically significant calculated by student “*t*” test

**Table-II:** Risk factors for HCV acquisition (n =343

	*Positive* *n = 146*	*Negative* *n = 197*	*Total* *n = 343*	*Odds ratio*	*95% confidence interval*	*P - Value*
*H/O Blood transfusion*						
Yes	102(69.9%)	106(53.8%)	208(60.6%)	1.99	1.26 – 3.12	0.004*
No	44(30.1%)	91(46.2%)	135(39.4%)			
*H/O therapeutic Injection use*						
Yes	124(84.9%)	137(69.5%)	261(76.1%)	2.46	1.43 – 4.26	0.001*
No	22(15.1%)	60(30.5%)	82(23.9%)			
*H/O Surgery*						
Yes	85(58.2%)	88(44.7%)	173(50.4%)	1.72	1.12 – 2.66	0.01*
No	61(41.8%)	109(55.3%)	170(49.6%)			
*H/O Dental consultation*						
Yes	32(21.9%)	31(15.7%)	63(18.4%)	1.50	0.86 – 2.60	0.16
No	114(78.1%)	166(84.3%)	280(81.6%)			
*H/O Sexual contacts*						
Yes	1(0.7%)	1(0.5%)	2(0.6%)	1.35	0.08 – 2.79	0.83
No	145(99.3%)	196(99.5%)	341(99.4%)			
*H/O Husband sexual contacts*						
Yes	7(4.8%)	21(10.7%)	28(8.2%)	0.42	0.17 – 1.02	0.07
No	139(95.2%)	176(89.3%)	315(91.8%)			
* H/O Drug abuses*						
Yes	10(6.8%)	11(5.6%)	21(6.1%)	1.24	0.51 – 3.01	0.65
No	136(93.2%)	186(94.4%)	322(93.9%)			
*H/O Sharing house holds*						
Yes	106(72.6%)	117(59.4%)	223(65.0%)	1.81	1.14 – 2.87	0.01*
No	40(27.4%)	80(40.6%)	120(35.0%)			
*H/O Piercing and tattooing*						
Yes	41(28.1%)	41(20.8%)	82(23.9%)	1.48	0.90 – 2.44	0.12
No	105(71.9%)	156(79.2%)	261(76.1%)			
*H/O HCV/HBV in family*						
Yes	40(27.4%)	42(21.3%)	82(23.9%)	1.39	0.84 – 2.29	0.20
No	106(72.6%)	155(78.7%)	261(76.1%)

*P value is statistically significant calculated by Fisher’s exact test.           H/O – History of

**Table-III:** Logistic regression analysis for HCV infection (n = 343

*Risk factors for hepatitis C*	*Adjusted Odds ratio*	*95% confidence interval*	*P - Value*
H/O Blood transfusion	0.743	0.41 – 1.34	0.323
H/O Injection use	0.545	0.29 – 1.02	0.059
H/O Surgery	0.846	0.48 – 1.46	0.550
H/O Dental consultation	0.879	0.49 – 1.57	0.665
H/O Sexual contacts	0.385	0.02 – 7.46	0.528
H/O Husband sexual contacts	2.603	0.97 – 6.97	0.05*
H/O Drug abuses	0.956	0.38 – 2.39	0.924
H/O Sharing house holds	0.654	0.39 – 1.08	0.097
H/O Piercing and tattooing	0.802	0.47 – 1.35	0.406
H/O HCV/HBV in family	0.942	0.55 – 1.60	0.827

*P value is statistically significant.           H/O – History of

## RESULTS

The frequency of HCV positive pregnant women in our study was 4.7%, out of 3078 obstetric admissions. Demographic details of cases and controls are shown in Table-I, without any statistically significant difference observed between parameters. Table-II shows measures of association between cases and controls with regard to various risk factors. HCV positive women were more likely to have history of (H/O) blood transfusion (OR 1.99, 95% CI 1.26- 3.12), H/O therapeutic injection use (OR 2.46, 95% CI 1.43-4.26), and H/O surgery (OR 1.72, 95% CI 1.12-2.66) and H/O sharing household items (OR 1.81, 95% CI 1.14-2.87).

Logistic regression was used to control the effects of various risk factors under study and to see the direct effects of a single risk factor on HCV positive status. Using logistic regression, no significant association was noted between all the variables & HCV positivity in pregnant women.

## DISCUSSION

In our study, seropositivity of HCV status among pregnant population was found to be 4.7% comparable to the nationwide figure of 3.3% to 29.1% reported in a review article by Umer and colleagues.^3^ In line with published literature, therapeutic injection use, blood transfusion and history of past surgical procedures were found to be leading risk factors for acquiring HCV infection.^6^^-^^8^

Therapeutic injections are routinely used in general practice in Pakistan, majority of which are unnecessary, unsafe and utilizes reused syringes. A survey conducted at Aga Khan University Hospital Clinics Karachi revealed that about half of the patients gave history of injection use at their last visit to health care providers and 3.5% of them had received 10 or more injections in the last year.^9^ Janjua & colleagues in their study from Sindh province reported an alarmingly high frequency of therapeutic injection use as 13.6 injections per person per year.^10^ Most of injections used were of uncertain sterility. It has been reported that many drug stores sell reused and repackaged unsterilized syringes, which could not be differentiated from sterilized syringes.^11^ Researchers are of the view that unsafe injection use is a major contributing factor for high HCV prevalence.^7^^,^^12^ Our study findings strongly support this view.

Pregnant women in Pakistan often require blood transfusion for the treatment of severe anaemia and life threatening complications like post partum hemorrhage. However it may endanger their later life by transmitting blood borne infections like HBV and HCV as evidenced by many researchers.^13^^,^^14^ Our study also highlights this fact. Blood transfusion is not at par with standard international guidelines in Pakistan, due to lack of proper screening and voluntary donors. Scarcity of organized infrastructure, standard operating procedures, lack of trained staff and non affordability of people to pay for screening particularly in rural areas are the main contributors for the spread of disease.^15^ To ensure safe blood transfusion, concrete efforts are needed on the part of government including promotion and recruitment of voluntary non-paid donors by creating public awareness, standardization and regulation of blood screening in blood banks at national level and judicious use of blood products by health care providers.

History of surgery including Caesarean section, laparotomy and D&C was another risk factor identified in our survey. Similar association was also reported by Jaffery T & Colleagues from Shifa International Hospital Islamabad in 2001 and 2002.^16^ As a matter of fact our survey population belongs to the lower socio-economic class who usually seek prenatal and postpartum care in the facilities where standard sterilization practices are not utilized both by qualified and unqualified staff which might be responsible for the spread of the disease.

Though the limitations of the study include being hospital based, but, as the LUH is a 1300 bed teaching hospital catering for the need of an enormous population within the radius of 250 km, its data gave an insight about prevalence of HCV infection in the population.

## CONCLUSION

In conclusion, unsafe and injudicious blood transfusion, therapeutic injection use and surgical intervention were found to be strongly associated with HCV infection. 

Concrete and comprehensive efforts are urgently needed by Pakistani government at all levels to control the spread of HCV infection. Besides promoting awareness in general public as well as health care providers, implementing preventing strategies in health facilities like use of screened blood transfusion, proper sterilization technique and use of disposable syringes will likely improve the worsening situation.
